# Supplementary material for: Effects of 36 hours of sleep deprivation on military-related tasks: Can ammonium inhalants maintain performance?
Source: PLoS One. 2023 Nov 15;18(11):e0293804. doi: 10.1371/journal.pone.0293804 (PMC10651003; doi:10.1371/journal.pone.0293804)
Supplement: S1 File — (PDF) [file pone.0293804.s003.pdf]

Formulář pro rozhodnutí EK *č.j.176 /20* F1

ROZHODNUTÍ ETICKÉ KOMISE

Název EK: ***ETICKÁ KOMISE NÁRODNÍHO ÚSTAVU DUŠEVNÍHO ZDRAVÍ***
Adresa EK: ***Topolová 748,250 67,Klecany***

Odpovídá složení EK požadavkům ICH GCP ? Ano [X] Ne [ ]

Pracuje EK podle jednacího řádu v souladu s předpisy ICH GCP? Ano [X] Ne [ ]

Datum a místo jednání : ***NUDZ odd.2 dne 16.9.2020 ve 13,30 hod.***

Jméno žadatele : ***Mgr. Kateřina Skálová***

Jméno / název zadavatele : ***Národní ústav duševního zdraví (NUDZ)***

Přesný název studie : ***„Okamžité účinky spánkové deprivace a amoniakových inhalačních prostředků na kognitivní a fyzickou způsobilost vojenského personálu“***. Ve spolupráci s FTVS UK.

Identifikační číslo datum protokolu : ***Viz.výše.***

Seznam hodnocené dokumentace :

Cover letter, Čestné prohlášení [X]
Složení řešitelského týmu, Synopse projektu [X]
IS a informace pro účastníky [X]
CV hl. řešitele [X]

Etická komise souhlasí s prováděním studie [X]

Projekt plně respektuje zásady Úmluvy o lidských právech a biomedicíně a zákon č.101/2000 Sb. o ochraně osobních údajů.

Etická komise nesouhlasí s prováděním studie [ ]

Důvody pro nesouhlas etické komise : ***0***

Požadavky etické komise : ***0***

Jednání etické komise se zúčastnili a hlasovali tito členové :

			Přítomen		Hlasoval	
			ANO	NE	ANO	NE
1.	Předseda :	Dr. Bareš	☒	☐	☒	☐
2.		Dr. Novák	☒	☐	☒	☐
3.		Mgr.Viktorinová	☐	☒	☐	☒
4.		Dr.KratochvílováMD	☐	☒	☐	☒
5.		Bc.Sobotka	☐	☒	☐	☒
6.		Bc.Švejdová	☒	☐	☒	☐
7.		pí.Švecová	☒	☐	☒	☐
8.		p.Kuneš	☒	☐	☒	☐
9.		Dr. Andrashko	☒	☐	☒	☐
10.		Dr. Hejzlar	☐	☒	☐	☒
11.		Bc. Baslová	☒	☐	☒	☐

Etická komise upozorňuje žadatele na jeho povinnost zaslat k posouzení etické komisi všechny dodatky protokolu před jejich provedením. Výjimkou jsou dodatky, které jsou určeny k eliminaci bezprostředních rizik pro subjekty hodnocení a ty dodatky, které jsou administrativního charakteru - tyto musí být následně ohlášeny etické komisi.
Dále musí žadatel předložit k posouzení komisi skutečnosti, které zvyšují riziko subjektů hodnocení nebo výrazně ovlivňují průběh studie, ohlásit komisi všechny zaznamenané závažné neočekávané příhody, ohlásit komisi nové informace, které mohou negativně ovlivnit bezpečnost subjektů hodnocení nebo průběh klinického hodnocení a podat komisi zprávu o průběhu klinického hodnocení, a to jednou ročně v jeho průběhu a dále po jeho ukončení. Tyto údaje se zasílají předsedovi etické komise

Datum : 16.9.2020

podpis předsedy: doc. MUDr. Martin Bareš, Ph.D.

Etická komise
Národní ústav duševního zdraví
Topolová 748, Klecany 250 67
tel.: 283 088 312
