# Supplementary material for: Effects of 36 hours of sleep deprivation on military-related tasks: Can ammonium inhalants maintain performance?
Source: PLoS One. 2023 Nov 15;18(11):e0293804. doi: 10.1371/journal.pone.0293804 (PMC10651003; doi:10.1371/journal.pone.0293804)
Supplement: S2 File — (PDF) [file pone.0293804.s004.pdf]

Decision form of the EC number.176/20

DECISIONS OF THE ETHICS COMMITTEE

Name of EC: ETHICAL COMMISSION OF THE NATIONAL INSTITUTE OF MENTAL HEALTH
Address of EC: **Topolová 748, 250 67, Klecany**

Does the composition of the EC meet the requirements of ICH GCP? YES [X] NO [ ]

Does the EC work according to the rules of procedure in accordance with ICH GCP regulations? YES [X] NO [ ]

Date and place of the meeting: NIHM 16.9.2020, 13:30

Name of applicant: Kateřina Skálová

Name of the contracting authority: National Institute of Mental Health (NIMH)

Name of study: "Immediate effects of sleep deprivation and ammonia inhalants on cognitive and physical fitness in military personnel". In cooperation with FTVS UK.

Identification number date of the protocol: See above

List of evaluated documentation:

Cover letter, Affidavit [X]
Leadership team composition, Project synopsis [X]
informed consent and information for participants [X]
CV of the principal investigator [X]

The Ethics Committee agrees to conduct the study [X]

The project fully respects the principles of the Convention on Human Rights and Biomedicine and Act No. 101/2000 Coll. on the Protection of Personal Data.

The Ethics Committee disagrees to conduct the study [ ]

Reasons for disagreement of the Ethics Committee: 0

Requirements of the Ethics Committee: 0

The following members of the Ethics Committee attended and voted:

			Present		Voted	
			YES	NO	YES	NO
1.	The Chair:	Dr. Bareš	X		X	
2.		Dr. Novák	X		X	
3.		Mgr. Viktorinová		X		X
4.		Dr. Kratochvílová		X		X
5.		Bc. Sobotka		X		X
6.		Bc. Švejdová	X			
7.		Ms. Švecová	X			
8.		Mr. Kuneš	X			
9.		Dr. Andrashko	X			
10.		Dr. Hejzlar		X		X
11.		Bc. Baslová	X			

The Ethics Committee reminds the sponsor of its obligation to send all amendments to the protocol to the Ethics Committee for review before they are made. The exceptions are those amendments which are intended to eliminate immediate risks to the subjects of the evaluation and those amendments which are of an administrative nature - these must be subsequently notified to the Ethics Committee. In addition, the sponsor had to submit to the committee for consideration the facts that increase the risk of the subject of the evaluation or significantly affect the course of the study, to report to the committee all recorded serious unexpected events, to report to the committee new information, that may adversely affect the safety of the subject or the conduct of the clinical trial, and to report to the committee on the conduct of the clinical trial, once a year during the trial and annually thereafter.

Date: 16.9.2020 The chairs' signature: doc. MUDr. Martin Bareš, Ph.D.

Etická komise
Narodní ústav duševního zdraví
Topolova 748, Klecany 250 67
tel.: 283 088 312
