# Supplementary material for: Effects of 36 hours of sleep deprivation on military-related tasks: Can ammonium inhalants maintain performance?
Source: PLoS One. 2023 Nov 15;18(11):e0293804. doi: 10.1371/journal.pone.0293804 (PMC10651003; doi:10.1371/journal.pone.0293804)
Supplement: S3 File — (DOCX) [file pone.0293804.s005.docx]

**Informovaný souhlas se zařazením do studie**

NÁZEV STUDIE: **„Okamžité účinky spánkové deprivace a amoniakových inhalačních prostředků na kognitivní a fyzickou způsobilost vojenského personálu“**

**Jméno subjektu:**

**Informace pro účastníka studie**

VÝZKUMNÝ TÝM: RNDr. Zdeňka Bendová, Ph.D., Mgr. Kateřina Skálová, Mgr. Jan Maleček, James J. Tufano, Ph.D., CSCS*D, Mgr. Kateřina Červená, Mgr. Kamila Weissová,

Vážená paní, vážený pane,

byl/a jste vyzván/a k účasti ve výzkumném projektu **„Okamžité účinky spánkové deprivace a amoniakových inhalačních prostředků na kognitivní a fyzickou způsobilost vojenského personálu“**.

Před tím, než se rozhodnete, zda se výzkumu zúčastníte, přečtěte si prosím pozorně následující informace. Dozvíte se v nich o použitých metodách, průběhu studie a jejím významu.

**Proč studii děláme?**

V dnešní společnosti dochází k nárůstu prací na směnný provoz, která výrazně zkracuje spánek a narušuje pravidelnost cirkadiánních rytmů a správnou funkci cirkadiánního systému člověka, jehož narušování je spojeno s vyšším rizikem rozvoje mnoha fyzických a duševních onemocnění. Neexistuje návrh na řešení problematiky k udržení kognitivních funkcí během noční směny a zároveň rychlé navrácení do fáze odpočinku, kdy by nedocházelo k narušování cirkadiánních rytmů.

V této studii bychom chtěli ověřit pomocí měření fyziologických parametrů, které jsou regulovány cirkadiánním systémem, dopad spánkové deprivace a na psychickou a fyzickou výkonost dobrovolníků z řad vojenského personálu.

**Jak bude studie probíhat?**

Pokud budete souhlasit s účastí ve studii, položí Vám výzkumník několik jednoduchých otázek ohledně Vašeho zdraví, spánkových zvyklostí a demografických charakteristik. Pokud v současné době užíváte nějaké léky, pokud jste prodělal/a určitá onemocnění, nebo pokud užíváte látky ovlivňující spánek, může se stát, že do studie nebudete moci být zařazen. Překážkou v účasti ve studii může být i to, pokud jste v průběhu 1 roku před konáním studie pracoval ve směnném provozu, či pokud jste nedávno cestoval přes více než 3 časová pásma. Po dobu jednoho týdne před začátkem studie Vás poprosíme zdržet se konzumace alkoholu, po dobu 48 hodin před hlavními experimenty prosíme vynechat kávu a jiné kofein obsahující nápoje.

Naše studie zabere čtyři setkání. Během první etapy - ,,Familiarization testing“, která bude probíhat týden před začátkem samotného experimentu, budete poučeni o průběhu studie, všemi testy a dodržování určitých zásad během experimentu na půdě Fakulty tělesné výchovy. Včetně účinků a způsobu inhalace uhličatu amonného (NH_4_)_2_CO_3,_ který je účinnou součástí přípravku Dynarex (kaple obsahuje 0,3 ml uhličitanu amonného v roztoku 35% etanolu) během kognitivních a fyzických testů. Přípravek využívají sportovci při trénincích či sportovních kompeticích. Při inhalaci přípravku dojde k okamžité aktivaci inhalačního reflexu, zrychlení dýchání, zvýšení srdeční frekvence a také napomáhá ke zvýšení bdělosti. Při dodržení všech předepsaných bezpečnostních předpisů (např. 15 cm vzdálenost aplikace od dýchacího ústrojí, dodržování doporučeného dávkování apod.) není látka nebezpečná pro zdravého jedince viz bezpečnostní list a vyjádření Etické komise UK FTVS.

Také budete vybaveni aktigrafem, přístrojem podobným hodinkám, určeným pro monitorování pohybové aktivity a intenzity okolního osvětlení. Aktigraf byste měli nosit nepřetržitě, pouze s výjimkou plavání a saunování, na zápěstí nedominantní ruky. Společně s aktigrafem obdržíte také senzor (čidlo) tělesné teploty (zařízení velikosti knoflíkové baterie s průměrem podobným korunové minci). Teplotní čidlo byste nosil připevněné na vnitřní straně bavlněného potítka tak, aby se dotýkalo zápěstí v místě, kde si lze nahmatat srdeční tep. Teplotní čidlo byste nosil nepřetržitě ve dne i v noci, stejně jako aktigraf, s výjimkou sprchování/koupání, plavání a saunování. Upevňování teplotního čidla je velmi snadné, jeho sundávání a opětovné nasazování Vám ukážeme. Nošení aktigrafu i teplotních čidel s sebou nenese žádná rizika. Obě zařízení budete nosit během studie i týden (dohromady 14 dní) po skončení pobytu ve spánkové laboratoři NUDZ. O jejich správném nošení budete znovu poučeni i během této etapy.

Během fáze ,,Baseline testing“ se dostavíte ve čtvrtek ve večerních hodinách do spánkové laboratoře NUDZ, kde zůstanete zde až do nedělního rána. Bude Vám přidělen vlastní pokoj a během spánku Vám budou nasezeny elektrody v podobě speciální čepice pro účel polysomnografického vyšetření. Polysomnografické vyšetření slouží k získání záznamu elektrické aktivity Vašeho mozku a dalších biosignálů (pohyby očí, napětí svalů, aktivita) v průběhu spánku. V rámci vyšetření se ke snímání používají elektrody, které se umístí na hlavu, na místa definovaná mezinárodními standardy – jedná se zejména o vlasatou část hlavy a také o čelo. Elektrody budou zabudované ve speciální čepici. Aby signál mohl být zachycen, je nutné pod každou elektrodu vstříknout vodivý gel nebo vodivou pastu. Gel i pastu lze snadno umýt.

V pátek v dopoledních hodinách proběhnou hlavní tři testy (střelba laserem, fyziologické a kognitivní testy), které Vám byly demonstrovány při první fázi. Po ukončení testování se dostavíte do sesterny, kde proběhne jednorázový odběr krevních vzorků (pro pozdější stanovení hladiny glukózy a kortizolu). Odběr krve je standardním zdravotnickým výkonem používaným v medicíně a množství odebrané krve nepřesáhne 3 ml a ani po opakovaných odběrech nepředstavuje žádné zdravotní riziko. Krevní odběry se uskuteční dvakrát denně po 12 hodinách, a to ve 10h a 22h.

Po odběrech začnete dle harmonogramu (10h, 14h, 18h, 20h, 22h, 24h, 02h, 04h, 06h, 08h, 10h) odebírat sliny do speciálních označených zkumavek. Je důležité vyvarovat se konzumaci jídla alespoň 30 min před jedním odběrovým bodem. Na odběr všech vzorků a správný průběh dohlédne výzkumný pracovník.

Během těchto odběrů zůstáváte v přiděleném pokoji i během začátku poslední fáze experimentu. Ta spočívá v absenci spánku po dobu následující noci a dne. Spánková deprivace bude probíhat pod vlivem konstantních světelných podmínek. Bude Vám umožněno využívat PC a zařízení s LED displeji. V ranních hodinách opět podstoupíte stejné testy jako předchozí den akorát pod vlivem spánkové deprivace. Poté bude opět proveden jednorázový krevní odběr.

Během dalšího dne zůstanete znovu ve svých pokojích a během noci Vám bude umožněn spánek, ale monitorovaný polysomnografickým vyšetřením (stejnou metodou jako během čtvrteční noci). Následující den podstoupíte ráno poslední kognitivní testování a dále budete propuštěni ze spánkové laboratoře. Aktigrafy a teplotní čidla Vás poprosíme nosit ještě po dobu jednoho týdne, abychom mohli monitorovat případné změny.

Během dalších 2-3 týdnů po ukončení našich experimentů budete opět pozváni do spánkové laboratoře, kde proběhnou opětovná měření ve všech parametrech a spánková deprivace během kontrolovaných temnostních podmínek. V pokoji, kde se budete během noci zdržovat, bude slabé oranžové či červené světlo (intenzita pod 2 lux). Během temnostní fáze Vám již nebude umožněno používat zařízení emitující modré světlo nebo přístroje s technologii LED displejů. Na průběh a dodržování bdělého stavu bude dohlížet jeden z výzkumných pracovníků. V případě opuštění místnosti budete opatřeni oranžovými brýlemi, které zabrání osvícení nežádoucím světlem. Studie končí opět v neděli v dopoledních hodinách po provedení kognitivních testů a po dobu jednoho týdne si opět ponecháte aktigrafy s teplotními čidly.

**Popis kognitivních a fyzických testů**

**Kognitivní testy**

Jedná se o počítačové testy, které mají za cíl měřit reakční čas a rozhodovací schopnosti. Před testováním střelby a výskoku podstoupíte 10 minutové kognitivní testování, které spočívá ve snaze reagovat co nejrychleji stiskem mezerníku na vizuální stimulus, který Vám bude v různých časových rozestupech promítán na monitor počítače. Během probdělé noci v rámci navození spánkové deprivace budete v různých časových intervalech podstupovat další podobné kognitivní testy na počítači, abychom zjistili změny parametrů v čase. Při spánkové deprivaci „ve tmě“ budou tyto testy prováděny s velmi slabým jasem monitoru a s nasazenými brýlemi blokujícími modré spektrum.

**Testování střelby laserem**

bude prováděno pomocí přenosné laserové střelnice. Během testování bude měřena přesnost střelby z makety služební zbraně s laserovým zaměřováním. Budete střílet do 3 různých terčů ve vzdálenosti 7 m od střelce. Vystřelíte celkem devětkrát (3 pokusy na každý terč). Poté se nadechnete čichací soli a zopakujete střelbu. Testování nepřekročí 10 minut.

**Countermovement jump (CMJ):** jde o skok, který měří výbušnou sílu. Postavíte se na silové desky (Kistler 141 9286BA, Kistler Instruments Inc, Winterthur, Switzerland) sloužící k měření inverzní dynamiky tj. reakci podložky na zatížení. Vaše postavení těla bude monitorovat lineární snímač polohy (Linear Positional Transducer, GymAware), který bude připojen k dřevěné tyči, kterou budete držet na ramenou. Provedete 3 CMJ a po inhalaci čichací soli dalších 6 skoků. Testování nepřekročí 10 minut.

**Důvěrnost údajů**

Pokud se studie zúčastníte, veškeré informace o Vás budou považovány za důvěrné. K informacím shromážděným v průběhu studie budou mít přístup odborní pracovníci Národního ústavu duševního zdraví a Etické komise Národního ústavu duševního zdraví v pseudoanonymizované formě. V žádné databázi nebude figurovat Vaše jméno. Vaše údaje budou zpracovávány a vedeny výhradně pod kódovým označeným. Propojení kódu s Vaším jménem může znát pouze výzkumní pracovníci.

Pokud budou výsledky studie prezentovány či publikovány v odborném tisku, bude to výhradně způsobem, aby nebylo možné určit žádné informace o konkrétním účastníku studie.

Svůj souhlas s použitím osobních údajů máte právo kdykoli odvolat zasláním písemného oznámení zkoušejícímu lékaři. Pokud svůj souhlas odvoláte, nebudete se moci studie nadále účastnit. Jestliže se však tak rozhodnete, nebudete nijak postižen/-a nebo znevýhodněn/-a v porovnání se situací před vstupem do studie.

**Povinnost účasti ve studii:**

Vaše účast ve studii je dobrovolná. Můžete odmítnout účast nebo můžete kdykoliv odstoupit bez udání důvodu, bez toho, že by to mělo vliv na péči, která je Vám poskytována. O ukončení účasti ve studii může rovněž rozhodnout zadavatel či Etická komise Národního ústavu duševního zdraví.

**Protokol studie schválila Etická komise Národního ústavu duševního zdraví.**

Máte-li jakékoliv dotazy ohledně samotné studie, obraťte se Mgr. Kateřinu Skálovou, mobil: 733640607, e-mail: [katerina.skalova@nudz.cz](mailto:katerina.skalova@nudz.cz)

**V případě dotazů ohledně etických aspektů výzkumu se můžete obrátit na jejího předsedu**

Etická komise NUDZ, e-mail: [ek@nudz.cz](mailto:ek@nudz.cz), tel. (+420) 283 088 312

NUDZ, lékař psychiatr – předseda EK: doc. MUDr. Martin Bareš, Ph.D.,

e-mail: [martin.bares@nudz.cz](mailto:martin.bares@nudz.cz), tel. (+420) 283 088 312

……………………………………. …………………………………….

Jméno a příjmení výzkumníka Datum, podpis

**Informovaný souhlas**

Svým podpisem stvrzuji, že jsem si přečetl/a výše uvedené informace, těmto informacím rozumím a dobrovolně souhlasím se svou účastí ve studii. Zároveň převezmu podepsaný stejnopis tohoto formuláře.

…………………………………. ……………………………………

Jméno a příjmení účastníka studie Datum, podpis
