# Supplementary material for: Effects of 36 hours of sleep deprivation on military-related tasks: Can ammonium inhalants maintain performance?
Source: PLoS One. 2023 Nov 15;18(11):e0293804. doi: 10.1371/journal.pone.0293804 (PMC10651003; doi:10.1371/journal.pone.0293804)
Supplement: S4 File — (DOCX) [file pone.0293804.s006.docx]

**Informed consent for inclusion in the study**

STUDY TITLE: **"Immediate effects of sleep deprivation and ammonia inhalants on cognitive and physical fitness of military personnel"**

**Name of the subject:**

**Information for study participant**

RESEARCH TEAM: RNDr. Zdeňka Bendová, Ph.D., Mgr. Kateřina Skálová, Mgr. Jan Maleček, James J. Tufano, Ph.D., CSCS*D, Mgr. Kateřina Červená, Mgr. Kamila Weissová,

Madam, sir,

You have been invited to participate in the research **project "The immediate effects of sleep deprivation and ammonia inhalants on the cognitive and physical fitness of military personnel"**. Please read the following information carefully before deciding whether to take part in the research. You will learn about the methods used, the study process and its significance.

**Why are we doing the study?**

In today's society, there is an increase in shift work, which significantly shortens sleep and disrupts the regularity of circadian rhythms and the proper functioning of the human circadian system, the disruption of which is associated with a higher risk of developing many physical and mental illnesses. There is no proposal to address the issue to maintain cognitive function during the night shift and at the same time quickly return to the rest phase when circadian rhythms are not disrupted.

In this study, we would like to verify the impact of sleep deprivation on the mental and physical performance of volunteer military personnel by measuring physiological parameters that are regulated by the circadian system.

**How will the study be conducted?**

If you agree to take part in the study, the researcher will ask you a few simple questions about your health, sleep habits and demographic characteristics. If you are currently taking any medications, if you have had certain illnesses, or if you are taking substances that affect your sleep, you may not be able to be included in the study. You may also be prevented from participating in the study if you have worked in shift work in the 1 year prior to the study or if you have recently travelled across more than 3 time zones. We will ask you to abstain from alcohol consumption for one week prior to the start of the study, and please abstain from coffee and other caffeine-containing beverages for 48 hours prior to the main experiments.

Our study will take four sessions. During the first stage, "Familiarization testing", which will take place a week before the actual experiment, you will be instructed on the study process, all the tests and the observance of certain rules during the experiment at the Faculty of Physical Education. Including the effects and method of inhalation of ammonium carbonate (NH4)2CO3, which is the active ingredient in Dynarex (a droplet containing 0.3 ml of ammonium carbonate in a 35% ethanol solution) during the cognitive and physical tests. The preparation is used by athletes during training or sporting competitions. When inhaling the preparation, there is an immediate activation of the inhalation reflex, acceleration of breathing, increase in heart rate and also helps to increase alertness. When all prescribed safety regulations are observed (e.g. 15 cm distance of application from the respiratory tract, observance of recommended dosage, etc.) the substance is not dangerous for a healthy individual, see the safety data sheet and the statement of the Ethics Committee of the UK FTVS.

You'll also be equipped with an actigraph, a watch-like device designed to monitor movement activity and ambient light intensity. The actigraph should be worn continuously, except for swimming and sauna use, on the wrist of the non-dominant hand. Along with the actigraph, you will also receive a body temperature sensor (sensor) (a device the size of a button battery with a diameter similar to a crown coin). You would wear the temperature sensor attached to the inside of a cotton sweatband so that it touches your wrist at the point where you can feel your heartbeat. You would wear the temperature sensor continuously day and night, just like an actigraph, except when showering/bathing, swimming, and taking a sauna. Attaching the temperature sensor is very easy, we will show you how to take it off and put it back on. Wearing both the actigraph and temperature sensors carries no risks. You will wear both devices during the study and for a week (14 days total) after your stay in the NUDZ sleep lab. You will again be instructed on their proper wearing during this phase.

During the "Baseline Testing" phase, you will report to the NUDZ Sleep Lab on Thursday evening and remain there until Sunday morning. You will be assigned your own room and during your sleep you will be fitted with electrodes in the form of a special cap for the purpose of polysomnographic testing. The polysomnographic examination is used to obtain a record of your brain's electrical activity and other biosignals (eye movements, muscle tension, activity) during sleep. The scan uses electrodes that are placed on the head, at locations defined by international standards - this includes the hair on the head and also the forehead. The electrodes will be embedded in a special cap. In order to capture the signal, a conductive gel or conductive paste must be injected under each electrode. Both the gel and the paste can be easily washed off.

On Friday morning, the main three tests (laser shooting, physiological and cognitive tests) that were demonstrated to you during the first phase will be performed. After testing, you will report to the nurses' station for a one-time blood sample collection (for glucose and cortisol levels later). The blood collection is a standard medical procedure used in medicine and the amount of blood collected will not exceed 3 ml and will not pose any health risk even after repeated collections. Blood draws will be performed twice a day for 12 hours each, at 10am and 10pm.

After the blood draws, you will start collecting saliva in special marked tubes according to the schedule (10h, 14h, 18h, 20h, 22h, 24h, 02h, 04h, 06h, 08h, 10h). It is important to avoid eating food at least 30 min before one collection point. A researcher will supervise all sampling and correct procedure.

You will remain in your assigned room during these collections and during the start of the last phase of the experiment. This involves the absence of sleep for the following night and day. Sleep deprivation will take place under constant light conditions. You will be allowed to use a PC and devices with LED displays. In the morning you will again undergo the same tests as the previous day, but under sleep deprivation. Afterwards, a single blood draw will be performed again.

During the next day you will stay in your rooms again and during the night you will be allowed to sleep but monitored by polysomnography (the same method as during Thursday night). The following day, you will undergo your final cognitive testing in the morning and continue to be discharged from the sleep lab. We will ask you to wear the actigraphs and temperature sensors for one more week so that we can monitor any changes.

During the next 2-3 weeks after the end of our experiments, you will be invited back to the sleep lab for repeat measurements in all parameters and sleep deprivation during controlled darkness conditions. The room you will be in during the night will have a dim orange or red light (intensity below 2 lux). You will no longer be allowed to use blue light emitting devices or devices with LED display technology during the dark phase. A member of the research staff will supervise the wakefulness process and compliance. If you leave the room, you will be provided with orange goggles to prevent illumination by unwanted light. The study ends again on Sunday morning after the cognitive tests have been completed and you will again keep the actigraphs with temperature sensors for one week.

**Description of cognitive and physical tests**

**Cognitive tests**

These are computerized tests that aim to measure reaction time and decision-making abilities. Prior to the shooting and jumping tests, you will undergo 10 minutes of cognitive testing, which consists of trying to react as quickly as possible by pressing the space bar to a visual stimulus that will be projected onto a computer monitor at various intervals. During the waking night, as part of inducing sleep deprivation, you will undergo other similar cognitive tests on the computer at different time intervals to detect changes in parameters over time. During sleep deprivation "in the dark", these tests will be performed with the monitor brightness very low and with blue spectrum blocking glasses on.

**Laser shooting testing**

will be conducted using a portable laser firing range. During the testing, the accuracy of the firing of the mock-up service weapon with laser sighting will be measured. You will be shooting at 3 different targets at a distance of 7 m from the shooter. You will fire a total of nine shots (3 attempts per target). You will then inhale smelling salts and repeat the firing. Testing will not exceed 10 minutes.

**Countermovement jump (CMJ):** this is a jump that measures explosive power. You stand on a force plate (Kistler 141 9286BA, Kistler Instruments Inc, Winterthur, Switzerland) used to measure inverse dynamics, i.e. the response of the pad to the load. Your body position will be monitored by a linear position transducer (Linear Positional Transducer, GymAware) which will be attached to a wooden bar that you will hold on your shoulders. You will perform 3 CMJs and 6 more jumps after inhaling smelling salts. Testing will not exceed 10 minutes.

**Confidentiality of data**

If you participate in the study, all information about you will be kept confidential. The information collected during the study will be accessible to the professional staff of the National Institute of Mental Health and the Ethics Committee of the National Institute of Mental Health in a pseudo-anonymized form. Your name will not appear in any database. Your data will be processed and kept only under a code. Only the researchers may know the association of the code with your name.

If the results of the study are presented or published in the professional press, it will be exclusively in such a way that no information about a specific study participant can be identified.

You have the right to withdraw your consent to the use of your personal information at any time by sending written notice to the investigating physician. If you withdraw your consent, you will no longer be able to participate in the study. However, if you choose to do so, you will not be affected or disadvantaged in any way compared to your situation before entering the study.

**Obligation to participate in the study:**

Your participation in the study is voluntary. You may refuse to participate or you may withdraw at any time without giving a reason, without affecting the care you receive. The sponsor or the Ethics Committee of the National Institute of Mental Health may also decide to stop participating in the study.

The study protocol was approved by the Ethics Committee of the National Institute of Mental Health.

If you have any questions about the study itself, please contact Mgr. Kateřina Skálová, mobile: 733640607, e-mail: katerina.skalova@nudz.cz

If you have any questions about the ethical aspects of the research, you can contact the chair of the committee.

NUDZ Ethics Committee, e-mail: ek@nudz.cz, tel. (+420) 283 088 312

NUDZ, psychiatrist - chairman of the EC: doc. MUDr. Martin Bareš, Ph.D.,

e-mail: martin.bares@nudz.cz, tel. (+420) 283 088 312

........................................... ...........................................

Name and surname of the researcher Date, signature

Informed consent

By signing this form, I certify that I have read the above information, understand the information and voluntarily agree to participate in the study. I also accept the signed copy of this form.

........................................ ..........................................

Name of study participant Date, signature
