# Supplementary material for: Effects of 36 hours of sleep deprivation on military-related tasks: Can ammonium inhalants maintain performance?
Source: PLoS One. 2023 Nov 15;18(11):e0293804. doi: 10.1371/journal.pone.0293804 (PMC10651003; doi:10.1371/journal.pone.0293804)
Supplement: Attachment — Submitted filename: Response to Reviewers.docx [file pone.0293804.s008.docx]

Dear Editor,

We are deeply appreciative of the insightful comments provided on our manuscript. The reviewers' constructive feedback was instrumental, guiding us in refining and enhancing our work. After incorporating these suggestions, we believe the manuscript has become significantly stronger, more comprehensive, and better positioned to contribute meaningfully to the field.

**Review Comments to the Author:**

**Reviewer #1:** The revision of the manuscript is satisfactory and the

authors have addressed all the questions raised by the reviewers.

Thank you very much.

**Reviewer #2: Minor revisions:**

Thanks for your insightful feedback on our paper. We recognize the significance of your points and we incorporated them in our revised version to improve its rigor.

1- Line 185: Clarify that the provided summary measures represent age,

height, and weight.

Thank you for pointing out the need for clarification in line 185. We revised the sentence to explicitly indicate that the summary measures are for age, height, and weight. The revised sentence is: “Eighteen healthy male military cadets (age = 24.1 ± 3.0 years, height = 181.5 ± 6.3 cm, weight = 79.3 ± 8.3 kg, 4.0 ± 0.9 total years of service, all measurements reported as mean ± SD) serving at the Military department of Charles University participated in this study.”

2- Line 344: Typographical error: Drop the second "W" from "Shapiro-Wilk

W test."

Thank you for spotting the typographical error. We corrected it to "Shapiro-Wilk test" in the revised manuscript.

3- Line 367: Use a lower case t in t-test.

Thank you for your suggestion. We changed "T-test" to "t-test" in the revised manuscript.

4- Results: For clarity, state that non-significant interaction effects were dropped from the models before testing main effects. Tables of these results would help readers better grasp the them.

Thank you for your suggestion to clarify the handling of non-significant interaction effects in our analysis. We have added the sentence "Non-significant interaction effects were excluded from the models prior to the examination of main effects." to the Results section (at line 350), to address this point. We believe this addition will enhance the clarity of our methodology.

Thank you also for suggesting the inclusion of tables to enhance readers' understanding of the results. While we acknowledge the value this would add, we have chosen to include all detailed tables in the supplementary materials available online. This approach allows interested readers to access all the specifics while maintaining the conciseness of the manuscript.

5- Define pbonf at its first appearance.

Thank you for pointing out the need to define “p_bonf_” at its first appearance. We have revised the text to clarify this term. The first sentence (at line 346) now reads: “When the ANOVA tests demonstrated a statistically significant condition × time (× time spans or × percentage difference) interaction or a statistically significant main effect for condition, time, time spans, or percentage difference, post-hoc comparisons of the mean differences were performed using the Bonferroni correction (p_bonf_).”
